# Supplementary material for: From sequence to enzyme mechanism using multi-label machine learning
Source: BMC Bioinformatics. 2014 May 19;15:150. doi: 10.1186/1471-2105-15-150 (PMC4229970; doi:10.1186/1471-2105-15-150)
Supplement: Additional file 2 — Java code of ml2db. Additional file ml2db_code.tar.gz contains the Java source code to run the multi-label machine learning experiments and save the results to database. The code’s Javadoc is included. [file 1471-2105-15-150-S2.zip › additional file 2/ml2db/utils/doc/uk/ac/standrews/utils/main/database/class-use/TableColumn.html]

Uses of Class uk.ac.standrews.utils.main.database.TableColumn


JavaScript is disabled on your browser.


- Overview
- Package
- Class
- Use
- Tree
- Deprecated
- Index
- Help

- Prev
- Next

- Frames
- No Frames

- All Classes

## Uses of Class uk.ac.standrews.utils.main.database.TableColumn

- Packages that use TableColumn

  | Package | Description |
  | --- | --- |
  |  |  |
  | --- | --- |
  | uk.ac.standrews.utils.main.database |  |
- - ### Uses of TableColumn in uk.ac.standrews.utils.main.database

    Methods in uk.ac.standrews.utils.main.database that return TableColumn

    | Modifier and Type | Method and Description |
    | --- | --- |
    |  |  |
    | --- | --- |
    | `static TableColumn` | DbUtils.`getEndTimeColumn()` A table column to store a timestamp for the end of an experiment |
    | `static TableColumn` | DbUtils.`getIdColumn()` get the definition for an "id" column of type auto-increment (which is primary key) |
    | `static TableColumn` | DbUtils.`getStartTimeColumn()` A table column to store a timestamp for the beginning of an experiment |
    | `static TableColumn` | DbUtils.`getTimeDownloadedColumn()` |
    | `static TableColumn` | DbUtils.`getTimestampColumn()` |

    Methods in uk.ac.standrews.utils.main.database that return types with arguments of type TableColumn

    | Modifier and Type | Method and Description |
    | --- | --- |
    |  |  |
    | --- | --- |
    | `java.util.Vector<TableColumn>` | Table.`getColumns()` Get the `TableColumn` list |

    Methods in uk.ac.standrews.utils.main.database with parameters of type TableColumn

    | Modifier and Type | Method and Description |
    | --- | --- |
    |  |  |
    | --- | --- |
    | `int` | TableColumn.`compare(TableColumn f1, TableColumn f2)` Compares two columns by their name, returns -1 if f1 comes alphabetically before f2, 0 if the two are equal and +1 if f2 comes alphabetically before f1. |
    | `int` | TableColumn.`compareTo(TableColumn f2)` Compares two columns by their name, returns -1 if this column comes alphabetically before f2, 0 if the two are equal and +1 if f2 comes alphabetically before this column. |
    | `boolean` | TableColumn.`equals(TableColumn anotherColumn)` Compares two columns by their name, returns true if the two columns have the same name, false otherwise. |

    Method parameters in uk.ac.standrews.utils.main.database with type arguments of type TableColumn

    | Modifier and Type | Method and Description |
    | --- | --- |
    |  |  |
    | --- | --- |
    | `void` | Table.`initialise(java.lang.String tableName, DbManager dbManager, java.util.Vector<TableColumn> columns)` |

    Constructor parameters in uk.ac.standrews.utils.main.database with type arguments of type TableColumn

    Constructor and Description

    `Table(java.lang.String tableName,
    java.util.Vector<TableColumn> columns,
    DbManager dbManager)`

    Construct a table for writing to: the columns definitions (name, data type) must be known.

- Overview
- Package
- Class
- Use
- Tree
- Deprecated
- Index
- Help

- Prev
- Next

- Frames
- No Frames

- All Classes
